# Sensory Island Task (SIT): A New Behavioral Paradigm to Study Sensory Perception and Neural Processing in Freely Moving Animals

**DOI:** 10.3389/fnbeh.2020.576154

**Published:** 2020-09-25

**Authors:** Dardo N. Ferreiro, Diana Amaro, Daniel Schmidtke, Andrey Sobolev, Paula Gundi, Lucile Belliveau, Anton Sirota, Benedikt Grothe, Michael Pecka

**Affiliations:** ^1^Division of Neurobiology, Department Biology II, Ludwig-Maximilians-Universität München, Munich, Germany; ^2^Department of General Psychology and Education, Ludwig-Maximilians-Universität München, Munich, Germany; ^3^Graduate School of Systemic Neurosciences, Ludwig-Maximilians-Universität München, Munich, Germany; ^4^Institute of Zoology, University of Veterinary Medicine Hannover, Hanover, Germany; ^5^Faculty of Medicine, Bernstein Center for Computational Neuroscience Munich, Munich Cluster of Systems Neurology (SyNergy), Ludwig-Maximilians-Universität München, Munich, Germany

**Keywords:** psychophysics, sensory feedback, chronic recording, go no-go, freely moving, sound localization, frequency discrimination, orientation selectivity

## Abstract

A central function of sensory systems is the gathering of information about dynamic interactions with the environment during self-motion. To determine whether modulation of a sensory cue was externally caused or a result of self-motion is fundamental to perceptual invariance and requires the continuous update of sensory processing about recent movements. This process is highly context-dependent and crucial for perceptual performances such as decision-making and sensory object formation. Yet despite its fundamental ecological role, voluntary self-motion is rarely incorporated in perceptual or neurophysiological investigations of sensory processing in animals. Here, we present the Sensory Island Task (SIT), a new freely moving search paradigm to study sensory processing and perception. In SIT, animals explore an open-field arena to find a sensory target relying solely on changes in the presented stimulus, which is controlled by closed-loop position tracking in real-time. Within a few sessions, animals are trained via positive reinforcement to search for a particular area in the arena (“target island”), which triggers the presentation of the target stimulus. The location of the target island is randomized across trials, making the modulated stimulus feature the only informative cue for task completion. Animals report detection of the target stimulus by remaining within the island for a defined time (“sit-time”). Multiple “non-target” islands can be incorporated to test psychometric discrimination and identification performance. We exemplify the suitability of SIT for rodents (Mongolian gerbil, *Meriones unguiculatus*) and small primates (mouse lemur, *Microcebus murinus*) and for studying various sensory perceptual performances (auditory frequency discrimination, sound source localization, visual orientation discrimination). Furthermore, we show that pairing SIT with chronic electrophysiological recordings allows revealing neuronal signatures of sensory processing under ecologically relevant conditions during goal-oriented behavior. In conclusion, SIT represents a flexible and easily implementable behavioral paradigm for mammals that combines self-motion and natural exploratory behavior to study sensory sensitivity and decision-making and their underlying neuronal processing.

## Introduction

Understanding how specific behaviors (reflexes, motor patterns, sensory representations, subjective perception, or cognitive functions) arise from neural processing is a primary goal of neuroscience. Pioneering research on sensory processing was based on observations of organisms and their innate behavior in their natural habitats (von Frisch, 1954; Tinbergen, 1963; Lorenz, 1981). This minimal-intervention approach laid the groundwork for the study of natural behavior during ethologically adequate sensory stimulation, yet left questions regarding the underlying neuronal mechanisms and brain circuits largely unanswered. In the last decades, experimental methods to study neural activity in awake and behaving animals have been increasing in number and complexity, providing previously unreachable insights into processing capabilities of neural populations. However, the great complexity of these techniques often requires highly controlled experimental conditions, which in turn limit their ecological relevance. Thus, they are prone to underestimate the dimensionality of neuronal processing (Gao and Ganguli, 2015; Krakauer et al., 2017).

A central evolutionary driving force acting on sensory systems is the processing of environmental cues in relation to self-motion: the interdependence of a motor action and the resulting modulation of sensory information is a fundamental aspect of both neural coding and decision making (Etienne et al., 1996; Ma and Jazayeri, 2014; Case et al., 2015), because this reciprocal interaction with the outside world allows for the continuous update of the “internal framework” within which the sensory inputs are interpreted (von Holst and Mittelstaedt, 1950; review: Campbell and Giocomo, 2018). Accordingly, substantial neural resources are dedicated to gathering and interpreting sensory information in relation to one’s own voluntary actions (Keller et al., 2012; Rancz et al., 2015; Vélez-Fort et al., 2018). A number of studies recently demonstrated the impact of movement on neuronal processing across sensory modalities, including somatosensation (Fu et al., 2014; Kerekes et al., 2017), vision (Chiappe et al., 2010; Niell and Stryker, 2010; Maimon et al., 2010; Dadarlat and Stryker, 2017; Clancy et al., 2019), and audition (Zhou et al., 2014; Schneider et al., 2014; for review see Schneider and Mooney, 2018). Likewise, multisensory co-modulation of the physical properties of the environment is crucial for inference and sensory object formation (Noppeney et al., 2008; Diehl and Romanski, 2014; Altieri et al., 2015; Atilgan et al., 2018) and, thus, highlights the importance of active task engagement of the experimental animals. This informational framework is highly plastic and subject to context-dependent modulation (Chabrol et al., 2015; Deneux et al., 2019).

However, despite the fundamental role of self-movement during goal-oriented behavior and the resulting multisensory co-modulation in complex sensory scenes, experimental investigations including these aspects are still underrepresented in the literature (Krakauer et al., 2017). While reports on psychophysical measurements involving decision-making are recently increasing (Carandini and Churchland, 2013; Saleem et al., 2018; The International Brain Laboratory et al., 2020), to this date, a flexible experimental paradigm to study sensory processing during goal-oriented behavior in freely moving animals is lacking. Here, we modified and expanded the existing concept of using closed-loop free navigation assays (Polley et al., 2004; Whitton et al., 2014). We present the Sensory Island Task (SIT), a novel experimental paradigm to study sensory processing of variable modalities during unrestricted self-movement in actively engaged animals that also allows for simultaneous neural recordings.

## Materials and Methods

In SIT, animals freely explore an arena in the presence of sensory background stimulation. They are trained to search for a hidden target island (a small circular sub-space in the arena, see below). Upon entering the target island, the background stimulus switches to the target stimulus. The animals are trained to report the detection of the target stimulus by staying at this position in the arena (i.e., within the target island). The position of the target island is altered in each trial and, thus, can only be found by detection of the change in sensory stimulation. A trial is considered correct when the animal stays within the target island for a specific duration (“sit-time,” typically 5–6 s). After a correct trial, a food reward is dropped in the arena via an overhead food dispenser. Trials have a time limit (typically 60 s) after which they are considered incorrect. Additionally, in some experiments (multi-island, see section “Results” for details), non-target islands were introduced simultaneously with the target island. These islands triggered a different change of stimulation than the target and no reward was provided when sit-time was achieved. This design of the task renders it a natural implementation of the NO-GO sensory change detection task, which are typically used in head-fixed experiments (Carandini and Churchland, 2013) and here is replaced by a sit-in-place condition.

### Animals and Housing: Gerbils

Here, SIT was used in two sensory modalities (auditory and visual) and in two species. Mongolian gerbils (*Meriones unguiculatus*) were used to probe auditory frequency discrimination and identification (aSIT_freq_) and sound source localization (aSIT_loc_) as well as visual orientation discrimination (vSIT_ori_). All procedures involving gerbils were approved in accordance with the stipulations of the German animal welfare law (Tierschutzgesetz) (AZ 55.2-1-54-2532-74-2016 and AZ 55.2-1-54-2532-70-2016). The animals were from the breeding colony of the Biocenter of the Ludwig-Maximilians University Munich. Animals were housed in groups of 3–4 individuals with 12 h light/dark cycles.

### Animals and Housing: Mouse Lemurs

Additionally, aSIT_freq_ was conducted with two gray mouse lemurs (*Microcebus murinus*). The non-invasive experiments were in accordance to the NRC Guide for the Care and Use of Laboratory Animals, the European Directive 2010/63/EU, and the German Animal Welfare Act. They were approved by the Animal Welfare Committee of the University of Veterinary Medicine and approved and licensed by the Animal Welfare Committee of the LAVES (AZ 33.19-42502-04-18/3050). The animals were from the breeding colony of the Institute of Zoology of the University of Veterinary Medicine Hannover. Maintaining and breeding were permitted by the Landeshauptstadt Hannover and the Landesamt für Verbraucherschutz und Lebensmittelsicherheit (LAVES; AZ 42500/1H).

### Setup and Stimulation During aSIT_freq_ and aSIT_loc_ With Gerbils

**FIGURE 1:**
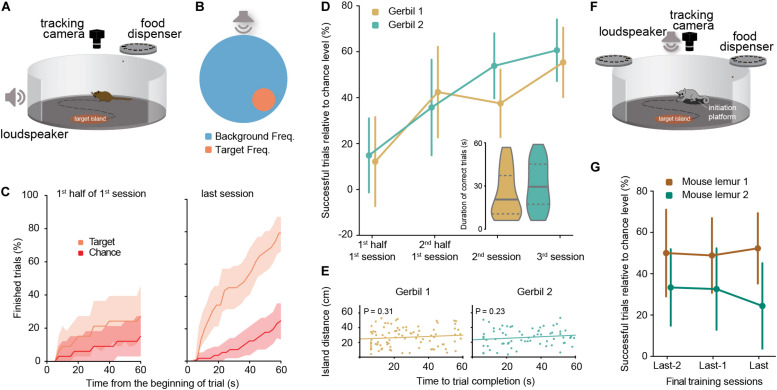
**(A)** Schematic of the experimental aSIT setup for gerbils. **(B)** Schematic representation (top view) of the aSIT_freq_ arena in the single island version for gerbils (background and target frequencies for gerbils were 20 kHz and 660 Hz). **(C)** Comparison for gerbil 1 of the percentage of trials finished with the percentage of trials which would have been finished by chance at each time point after the beginning of a trial (shadow areas correspond to 95% confidence interval); Left panel: 1st half of the 1st session; Right panel: 3rd session. **(D)** Percentage of successful trials relative to the chance level (as calculated in **C** at 60 s) for each gerbil (error bars correspond to the 95% confidence interval). Session 1: N_Gerbil 1_ = 66 trials, N_Gerbil 2_ = 55 trials; Session 2: N_Gerbil 1_ = 72 trials, N_Gerbil 2_ = 65 trials; Session 3: N_Gerbil 1_ = 56 trials, N_Gerbil 2_ = 61 trials. Inset: duration of successful trials for each gerbil in the two last training sessions, horizontal lines denote median (solid) and quartiles (dashed) of the distribution. Durations of correct trials per session are available in Supplementary Figure S2 (both for gerbils and for mouse lemurs). **(E)** Time to success in two consecutive successful trials was not correlated with geometric island distance in either gerbil. Pearson correlation, N_Gerbil 1_ = 89 pairs of trials, N_Gerbil 2_ = 68 pairs of trials. **(F)** Schematic of the experimental aSIT setup for mouse lemurs. Background and target frequencies for lemurs were 10 and 4 kHz, respectively. **(G)** Performance of two mouse lemurs in three consecutive days at the end of the training: percentage of successful trials relative to the daily chance level (as calculated in C; error bars correspond to the 95% confidence interval). Session 1: N_Lemur 1_ = 32 trials, N_Lemur 2_ = 48 trials; Session 2: N_Lemur 1_ = 43 trials, N_Lemur 2_ = 43 trials; Session 3: N_Lemur 1_ = 44 trials, N_Lemur 2_ = 41 trials. For performance levels during intermediate training sessions (see Supplementary Figure S3).

The aSIT_freq_ and aSIT_loc_ (freq: sound frequency as target indicator; loc: sound source location as target indicator) tests with gerbils were conducted in a custom-made setup consisting of a circular arena (diameter = 92 cm) within a sound attenuated chamber (Figure 1A). The arena floor consisted of a black-painted wood or PVC surface surrounded by perforated metal walls (height: 16 cm). Additionally, PVC walls were mounted on top of the metal wall around the entire arena up to a height of 75 cm.

**FIGURE 2:**
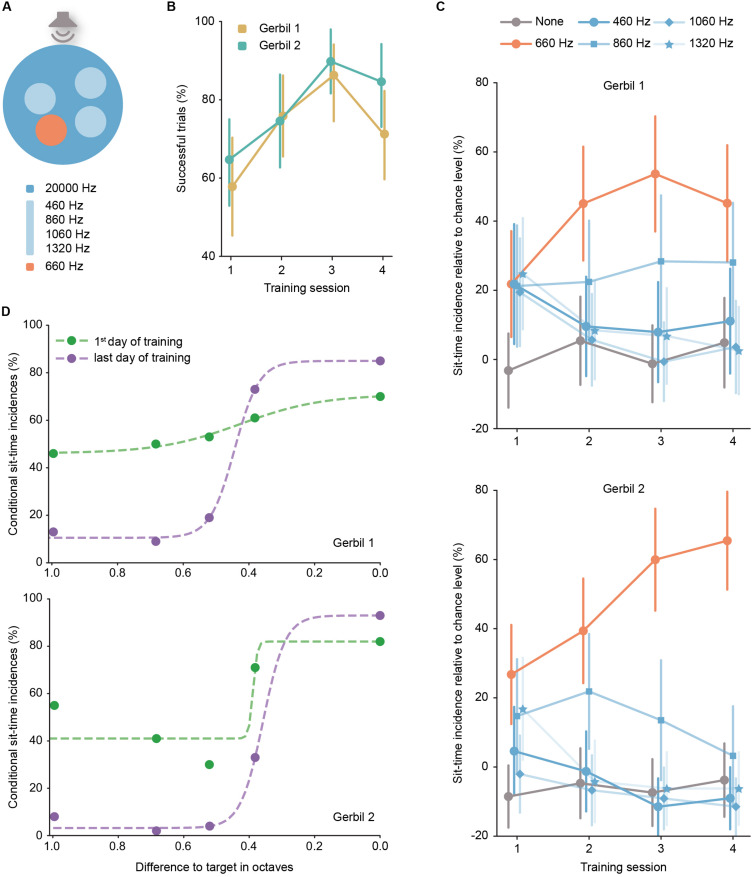
**(A)** Schematic representation (top view) of the aSIT_freq_ in the multiple island version. Note that on a given trial, only three of the four possible non-target frequencies were offered. **(B)** Performance of each gerbil per training session (error bars correspond to the 95% confidence interval). Session 1: N_Gerbil 1_ = 64 trials, N_Gerbil 2_ = 68 trials; Session 2: N_Gerbil 1_ = 58 trials, N_Gerbil 2_ = 59 trials; Session 3: N_Gerbil 1_ = 51 trials, N_Gerbil 2_ = 49 trials; Session 4: N_Gerbil 1_ = 62 trials, N_Gerbil 2_ = 52 trials. **(C)** Incidence of sit-time across sessions, relative to chance level per island (error bars correspond to the 95% confidence interval). **(D)** Psychometric function: comparison between the first and last training session of the percentage of events the animal stayed the sit-time in each island depending on the frequency distance in octaves of the island to the target frequency; results were fit with a logistic function (dashed line).

Stimuli were computer generated and transmitted through an amplifier (AVR 445 Harman/Kardon, Germany). Stimulus presentation was delivered through loudspeakers (Aurasound NSW1-205-8A 1″ Extended Range) mounted externally of the arena (∼5 cm distance to the metal walls). Auditory stimuli during aSIT_freq_ were 57 ms long pure tones with frequency according to task structure. Trial initiation elicited the playback of the background frequency (20 kHz), and animal entrance into an island triggered the switch of the frequency played to the target frequency of 660 Hz or non-target frequencies of 460, 860, 1060, or 1320 Hz (Figure 2 - see section “Results” for details). Stimuli during aSIT_loc_ were 57 ms long harmonic complex sounds with a fundamental frequency of 147±4 Hz and low-pass filtered below 1.5 kHz. Trial initiation triggered the playback of the above-mentioned harmonic complex by the background loudspeaker, and animal entrance into the island triggered the switch of the playback to the target loudspeaker. Stimuli in either aSIT version were played at a repetition rate of 4 Hz and their amplitude was 70 dB SPL roved ±5 dB, which rendered a stimulation of about 55 dB above background noise. The animal’s position was tracked via images captured every 250 ms with a Flea3 camera (FL3-U3-13Y3M-C, Point Grey Research Inc.), centered over the arena at a height of 130 cm from the arena floor. Stimulation parameters (i.e., sound frequency or source location) were updated online according to the animals’ position within the arena (see section “Results” for details). Custom-made software for animal tracking, stimuli generation and food reward delivery was developed in MATLAB. A custom-made overhead rotating food dispenser positioned 100 cm over the arena was used for automatic reward administration by dropping a food pellet (∼20 mg, TestDiet LabTab AIN-76A) or part of a sunflower seed after every correct trial. If the animal did not correctly report the target island within the time limit, a low-pass filtered noise was presented to the animal for 10 s, during which no new trial could be initiated.

### Behavioral Training During aSIT_freq_ and aSIT_loc_ With Gerbils

Two gerbils were used for the behavioral testing of the aSIT_freq_ paradigms, and 11 gerbils were tested in the aSIT_loc_ version of the task. Training of gerbils began at a minimum of 8 weeks of age. All gerbils within this study were male. Water and food (pellets) were provided *ad libitum* until training started, at which point food was only available during training sessions as reward for correct trials. No more than two training sessions were carried out per day, lasting up to 60 min each for aSIT_freq_ and up to 90 min for aSIT_loc_. Final parameters of island size (diameter = 25 cm, ∼7% of the arena surface) and sit-time (6 s) were identical for both aSIT_loc_ and aSIT_freq_. For aSIT_freq_, animals were presented with the final parameters from the beginning of training. For aSIT_loc_ the training of the animals was performed by gradually reducing island size (starting at diameter = 42 cm, ∼21% of the arena surface) and increasing sit-time (starting with 2 s) over the course of the training sessions. Additionally, for aSIT_loc_, animals were initially trained in a protocol with one slightly elevated, peripheral, circular initiation platform (diameter = 12 cm), which the animals had to visit in order to initiate a trial. For aSIT_freq_, an additional configuration with multiple islands was tested, where three non-target islands were available in the arena alongside the aforementioned target island (see section “Results” for details). All gerbils in the aSIT tasks underwent a general habituation period in the SIT setup for 15 min per day for 5 days.

### Setup and Stimulation During aSIT_freq_ With Mouse Lemurs

The aSIT_freq_ experiments with mouse lemurs were conceptually identical, yet with adapted parameters to accommodate to species-specific exploration behaviors. Experiments were conducted in a circular open field arena with a diameter of 80 cm and a height of 70 cm (Figure 1F). For online animal tracking, a camera (Logitech C500 Webcam) with removed infrared filter was positioned above the center of the maze and at a distance of 92 cm from the floor plate, so that the arena floor optimally fitted the vertical dimensions of the video picture. For acoustic stimulation, a single broadband speaker (Visaton B200, VISATON GmbH & Co., KG, Haan, Germany) was mounted above the arena at a distance of 165 cm from the arena floor. The floorplate was made of frosted light-conducting acrylic glass (Plexiglas^®^ LED, Evonik Industries, Darmstadt, Germany) and illuminated with infrared diodes (peak wavelength at 940 nm) from below to provide optimal contrast between background and experimental animal during tracking. The sidewall of the circular arena was made of opaque, dark-gray acrylic glass (Zimmermann + Collegen Kunststoff-Technik GmbH, Hannover, Germany). Food rewards used as positive reinforcement during the learning experiments (see below) were provided on-top of the regular, *ad libitum* diet. To provide food rewards (small peanut pieces of approximately 15 mg) for correct behavioral responses during training, commercially available aquarium feeders (Rondomatik 400, Grässlin GmbH, St. Georgen, Germany) were modified to be controllable with Arduino Uno microcontrollers via Arduino Uno motor shields (v1). Two of these modified feeders were installed at opposing positions on the arena wall (i.e., at a distance of approximately 70 cm from the floor) and their positions could easily be shifted between sessions to reduce predictability of the reward location. For online animal tracking as well as sound stimulation and hardware control based on the animal’s behavior, we used self-coded Python scripts, running on windows machines with Windows 7 and Python 3.7.

### Behavioral Training During aSIT_freq_ With Mouse Lemurs

Training of mouse lemurs was conducted in male individuals aged 5 and 6 years that had previously participated in non-auditory behavioral experiments unrelated to SIT. To avoid stress, subjects were transported to the setup in their sleeping boxes and experiments were conducted under low-light conditions (1–5 lux). Each animal was trained once per day during workdays in a single session of 60 min or 50 completed trials (depending on which limit was reached first). Animals were trained in a protocol with one slightly elevated, peripheral, circular initiation platform, which the respective animal had to visit in order to initiate a trial, and one circular target island. Once a trial had been initiated, a background sound (pure tone of 10 kHz, 57 ms duration, sound pressure level = 67.5 ± 2.5 dB) was played back at a repetition rate of approximately 5 Hz while the geometric center of the animal remained outside of the target island (pseudo-randomly generated position without overlap with the initiation platform). As soon as the animal entered the target island during a given trial, stimulation switched to the target sound (pure tone of 4 kHz, all other properties were identical to the background sound). The frequency of the stimuli was chosen to lie within the range of optimal hearing described for mouse lemurs (Schopf et al., 2014). If the animal failed to find the target island or to remain within it for the desired sit-time within a pre-defined trial duration, the trial stopped, as did the acoustic stimulation, and the animal had to revisit the initiation platform to start a new trial. During the experiments, the setup was illuminated with dim red light, comparable to the illumination of the housing rooms during the daily activity phase of the nocturnal mouse lemurs. While the location and size (diameter = 18 cm, 5% of arena surface) of the initiation platform were fixed values, the size of the target island, the sit-time, and the trial duration could vary between sessions. In the first session, the size of the target island was set to a diameter of 32 cm (∼16% of arena surface), the target duration to 1 s, and the trial duration to 120 s. To increase the difficulty with increasing training and to better differentiate behavioral responses to the target sound from chance-level performance, these variables were changed between sessions, depending on the animal’s performance on the preceding training days. Values for the final sessions were a target island diameter of 24 cm (∼9% of arena surface) and a sit-time of 5 s. Animals were trained until performance in three consecutive sessions under these conditions was above chance level.

### Setup and Stimulation During vSIT_ori_ With Gerbils

**FIGURE 3:**
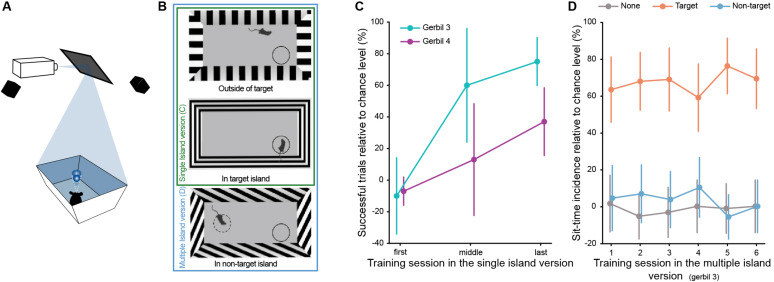
**(A)** Schematic representation of the ratCAVE setup (adapted from Del Grosso et al., 2017). **(B)** Schematic representation (top view) of the vSIT_ori_ arena, both in the single and in the multiple island version. Animal entrance to a target island and non-target island triggered the change of grating orientation from vertical to horizontal or oblique, respectively. All gratings used only differed in orientation angle. Differences in appearance is due to visual angle from above. **(C)** Performance of each gerbil in example sessions from the beginning, middle, and end of the training, in the single island task (error bars correspond to the 95% confidence interval). First session: N_Gerbil 3_ = 20 trials, N_Gerbil 4_ = 42 trials; middle session: N_Gerbil 3_ = 12 trials, N_Gerbil 4_ = 20 trials; last session: N_Gerbil 3_ = 40 trials, N_Gerbil 4_ = 32 trials. **(D)** Incidence of sit-time across sessions relative to chance level per island, in the multiple island task (error bars correspond to the 95% confidence interval). Gerbil 3 N_Session 1_ = 34 trials; N_Session 2_ = 41 trials; N_Session 3_ = 43 trials; N_Session 4_ = 39 trials; N_Session 5_ = 44 trials; N_Session 6_ = 39 trials.

The vSIT_ori_ experiments were conducted in a 3D virtual reality setup called ratCAVE (Del Grosso et al., 2017), which was designed for behavioral experiments in freely moving animals. To this end, a large rectangular arena (dimensions 162 cm × 72 cm and walls of 60 cm height, placed with a 70 degrees angle to accommodate the visual projection), was used. A set of 7 cameras (Prime 13W 240 fps, OptiTrack, NaturalPoint Inc., United States) served to record the 3D position of reflective markers fixed on the head of the animal. A projector with 240 fps frame rate (VPixx Technologies Inc., Canada), mounted to the ceiling, was used to project the image of the virtual environment on the walls of the arena depending on animal position (Figure 3A). A food dispenser (Campden Instruments Ltd.) positioned above the arena served for automatic reward administration by dropping a food pellet (∼20 mg, TestDiet LabTab AIN-76A) after every correct trial. A custom-written python-based software was used to manage the projection, animal rewarding, positioning, and data logging.

The virtual environment for the vSIT_ori_ experiment consisted of black and white square-wave grating patterns with stripes of 10 cm width, projected on all four walls of the arena. When animals entered the target island, the projected grating pattern on the walls changed its orientation from vertical to horizontal (Figure 3B). A non-target island was additionally implemented for one of the animals which, upon animal entrance, triggered change from the vertical grating projection to oblique (45 degrees). Each successful trial was followed by an inter-trial period of 15 s with only light projected on the arena floor (no patterns on the walls) to allow the animal to find the rewarded pellet. After the inter-trial interval, the new trial started automatically.

### Behavioral Training During vSIT_ori_ With Gerbils

Two male gerbils were trained in this version of vSIT_ori_. No habituation was required, as they had previously participated in another study within the same arena. Animals were food restricted and kept at a minimum weight of 85% of the *ad libitum* condition. Similar to the aSIT_loc_ experiments, training of the animals was performed by gradually reducing island size (starting at ∼10% of the arena surface) and increasing sit-time (starting at 2 s) over the course of the training sessions. At the end of the training (15 and 24 sessions), a trial was considered correct when the animal stayed within the target island of minimal size (∼6% of arena surface area) for a sit-time of 6 s. For one of the gerbils, the non-target island was introduced to the trials after performance reached a level significantly different from chance (see section “Results”).

### Source Code Availability

Protocols to perform aSIT_freq_ experiments are freely accessible for download at https://gin.g-node.org/asobolev/runsit/.

### Surgical Procedures and Chronic Electrophysiological Recordings

One adult male Mongolian gerbil (∼70 g) that was trained in aSIT_loc_ underwent tetrode implantation surgery. At the beginning of the surgery, the animal was anesthetized with an intraperitoneal injection of a mixture of metedomidin (0.15 mg/kg), midazolam (7.5 mg/kg), and fentanyl (0.03 mg/kg). The depth of the anesthesia was verified by lack of paw pinch or eye lid reflexes. To maintain it at a constant level, the same mixture was subcutaneously re-injected every 90 min. After shaving and disinfecting the head, a local anesthetic (50 μl, 2% xylocaine) was injected under the scalp skin and below the skin near the ears. For protection and to prevent dehydration, the eyes were covered with an ophthalmic gel (Thilo-Tears SE, Alcon Pharma GmbH). The animal was then transferred to the stereotactic apparatus, where its head was securely fixed via a bite and ear bars. Its internal temperature was monitored with a rectal thermometer and kept constant at 37°C throughout the experiment by a feedback controlled electric heating pad (Harvard Apparatus). After disinfection, a midline scalp incision was performed to expose the skull. Subsequently, the connective tissue on the skull was removed with a bone curette and the skull was treated with 35% phosphoric acid (iBOND etch gel, Kulzer), which was promptly washed away. Structural screws were placed on top of the left frontal and right parietal bones and the ground screw on the occipital bone, so that it gently touched the brain. After stereotactic alignment, a 3 × 3 mm craniotomy and durotomy were performed on top of the left auditory cortex, followed by a very slow lowering (2 μm/s) of a tetrode bundle to a maximum depth of 0.9 mm into the cortex, using a micromanipulator (Scientifica). The craniotomy was carefully filled with KY-jelly and immediately sealed with dental cement (Paladur, Kulzer), which also fixated the bottom of the microdrive and the outer cannula that protected the tetrodes. 1 ml of Ringer’s solution was subcutaneously injected at the end of the surgery and the anesthesia was reversed via subcutaneous injection of the antagonist mixture composed of naloxone (0.5 mg/kg), flumazenil (0.4 mg/kg), and atipamezol (0.375 mg/kg). Analgesics (0.2 mg/kg, meloxicam) and antibiotics (7.5 mg/kg, enrofloxacin) were orally administered post surgically for five subsequent recovery days. During this time, the animals had food and water *ad libitum* and were not trained.

**FIGURE 4:**
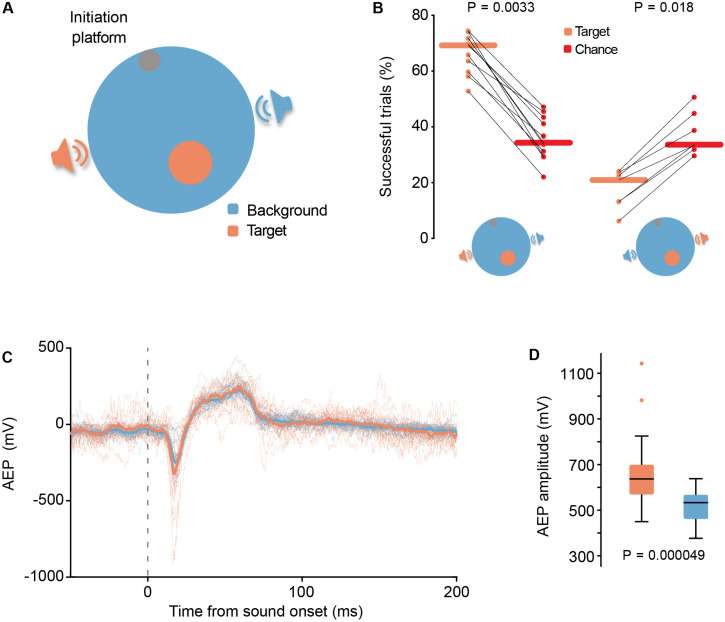
**(A)** Schematic representation (top view) of aSIT_loc_, a sound source localization version of SIT. **(B)** Left-hand panel: Reporting of sound location was highly significant (*P* = 0.0033, *N* = 11 gerbils, Wilcoxon signed-rank test) compared to their chance level given their actual locomotion behavior, calculated by surrogate island computation. Right-hand panel: In 1/8 of the trials the identity of the target and background loudspeakers was swapped for a subset of the animals. A significant decrease in the performance for the “swapped” trials below chance level (*P* = 0.018, *N* = 7 gerbils, horizontal lines depict the median, Wilcoxon signed-rank test) suggests the animals were actively avoiding the target island under these conditions. **(C,D)** Response magnitude differences in auditory evoked potential (AEP) recordings of auditory cortex neural populations. **(C)** Single session example traces. Dotted lines represent AEP per trial and active loudspeaker identity. Thick traces represent the median of all trials. **(D)** Quantification of AEP amplitude. In this example session the AEP amplitude was significantly larger during target loudspeaker activity (*P* = 0.000049; N_target_ = 19, N_background_ = 23; Mann-Whitney *U*-test). Boxplots depict the median (black line), 1st and 3rd quartile (filled boxes), ± 2.7 σ (whiskers) and outliers (crosses).

The implant used in this experiment was a tetrode bundle consisting of four tetrodes glued together, which, on their turn, consisted of four insulated tungsten wires (12.7 μm diameter each, tungsten 99.95%, California Fine Wire) twisted around each other. Each wire was connected to a custom-made printed circuit board with Omnetics connector (Axona), which was attached to a lightweight microdrive (0.25 mm/turn, Axona). The tetrodes were glued together and protected by an inner and outer cannula that could slide by each other. On the day prior to the surgery, the tip of all electrodes were cut with sharp scissors and gold plated (Non-Cyanide Gold Plating Solution, Neuralynx) to reach a desired impedance of 100–150 kOhm (at 1 kHz). The tetrode bundle was implanted vertically in the following coordinates from lambda: 6.2 mm lateral, 2.6 mm anterior. The recording depicted in Figure 4 occurred at an electrode depth of ∼1.3 mm.

Recorded signals were amplified and digitized (16-bit resolution) in the wireless headstage (W2100-HS16, Multichannel Systems), and transmitted to the receiver. Through an interface board (W2100-System, Multichannel Systems), the signal was then sent to the computer where it was acquired with a sampling rate of 25 kHz via commercial software (Multi Channel Experimenter). A digital signal for posterior alignment of the sounds and video with the neural signal was simultaneously sent to the interface board.

### Data Analysis

All data analyses were performed in MATLAB (Mathworks) and Python using custom scripts. To test the performance of the animals, we compared the percentage of correct trials in each session with surrogate runs based on random target island shuffling. That is, for each trial (offline, *a posteriori*), 1000 surrogate (non-real) islands, non-overlapping with the target one, were randomly set and the real trajectory of the animal was used to calculate in how many of these islands the trial would have been correct given the required sit-time (Supplementary Figure S1). At each time point, we determined how many trials were already finished and the respective uncertainty (95% confidence interval) was calculated based on bootstrapping (random sampling with replacement from all the trials of the session). The median chance performance and confidence interval at each time point was calculated based on bootstrapping from the random target island shuffling data (random sampling with replacement from the 1000 surrogate trials with number of trials as size of the sample). The chance performance calculation was based on trajectories from trials which were incomplete up to the considered time point (real target island not yet found) and trajectories in which the animal stayed longer than the sit-time in the surrogate island before that time point. A trial which had been finished by that time point and in which the animal did not find the surrogate island cannot be used in the bootstrapping of the time points posterior to the finishing time because it is unknown whether the animal would have found the island if the trial had been longer. This method allows obtaining an estimate of the proportion of correct trials the animal would have gotten just by chance given their locomotion trajectory and dynamics.

In the multi-islands version of SIT, the sit-time incidence was calculated by assigning an island to each trial. This assignment corresponds to the first island in which the animal stayed longer than the sit-time. For example, if the animal correctly finished a specific trial but had been sitting for longer than the sit-time in a non-target island prior to finishing, this trial is assigned to the respective non-target feature and not to the target one, even though the animal also remained sufficiently long in the target island later. A trial in which the animal never remained for longer than the sit-time in any island is assigned to “None.” In the aSIT_freq_ multi-island configuration, the target frequency is always present but the non-target frequencies are not, as there are 4 non-target frequencies and only 3 non-target islands in each trial. Therefore, for each session, we calculated the percentage of trials assigned to each frequency, normalized by the total amount of trials in which the respective frequency was available. As a measure of uncertainty, the 95% confidence interval was calculated by bootstrapping (the percentage calculation was done on 1000 random samples with replacement from the assignment to each frequency with number of trials as size of the sample). The chance level (calculated per animal with data from the last session of the single island version) was subtracted from this percentage and the 95% confidence interval was calculated using error propagation.

For the construction of the psychometric function, in each session all the events in which the animal stayed at least 1s in the island were identified. For those events, the percentage of times the animal stayed in a specific frequency island for the designated sit-time (6 s) was calculated. This allows the construction of a perception curve by fitting a logistic function $\frac{m\,a\,x}{1+e^{-s\,l\,o\,p\,e\,\left(x-x_{0}\right)}}+o\,f\,f\,s\,e\,t$ to these percentage values, with the frequency distance in octaves of each island to the target frequency as *x*; the offset in relation to zero describes the recurrent behavior of stopping randomly, which occasionally can last longer than the sit-time.

For the analysis of the local field potential (LFP), the recorded signal was low-pass filtered at 600 Hz. Auditory evoked potential (AEP) was calculated per trial, by loudspeaker active. Amplitude of the AEPs was calculated from peak to peak, that is, the difference between the maximum and minimum voltage recorded in the time window corresponding to the first 100 ms after stimulus onset.

### Statistics

Binomial tests were used to compare, on a given experimental session, the percentage of correct trials with the ones expected by chance, as calculated using the surrogate runs analysis.

All error bars correspond to the 95% confidence interval as calculated via bootstrapping, except for the boxplots in Figure 4D.

For the investigation of possible linear relationships between the distance between islands in consecutive trials and the time to completion in the latter trial (Figure 1E), we used Pearson correlation analyses.

For comparisons of central tendencies on the group level, we used two-tailed non-parametric tests: Wilcoxon signed-rank tests for paired samples and Mann-Whitney *U*-test for independent samples.

All hypotheses were tested at an alpha level of 0.05.

## Results

The Sensory Island Task (SIT) is an operant conditioning foraging task in an open-field arena (Figure 1). We designed SIT to allow for high flexibility regarding the implementation of sensory modalities and parameters to address the desired specific research question. Animals can roam freely in the arena, in search for a sensory “target island” (in auditory versions of SIT, we used a circular target area within the arena, area ∼5–9% of the arena surface), relying solely on changes in the presented stimulus, which is controlled in real-time via closed-loop position tracking. They are trained via positive reinforcement to discover the target island by detecting a change in stimulation from a “background” to a “target” stimulus. Animals report this detection of the target stimulus by remaining within the island for a defined time (sit-time). Upon correct reporting, a food reward is administered by dropping from an overhead dispenser, which ensures that any association of the reward consumption with a specific location in the arena is prevented (since the reward bounces unpredictably on the arena floor). The location of the target island is randomized across trials, making the stimulus feature under investigation the only informative cue for task completion. Multiple “non-target islands” (areas where the relevant stimulus feature is changed into neither the target nor the background and where the animal is not rewarded) can be incorporated in SIT to test identification performance. Furthermore, SIT can readily be adapted to the species and sensory system under investigation. To demonstrate this high flexibility, here we present data from Mongolian gerbils (*Meriones Unguiculatus*, rodents) and gray mouse lemurs (*Microcebus murinus*, small primates) trained in SIT to perform auditory frequency discrimination and identification (aSIT_freq_). We further demonstrate the suitability of SIT to study sound source localization (aSIT_loc_), as well as visual orientation identification and discrimination (vSIT_ori_).

### Auditory Frequency Discrimination (aSIT_freq_)

We trained animals to detect a change in the presented stimulus frequency upon entering the target island. Throughout a trial, a “background” frequency was played in repetitive pulses (duration 57 ms, repetition rate 4 Hz in rodents, 5 Hz in mouse lemurs) through a single loudspeaker as long as the animal was outside of the target island. Once (and if) the animal entered the target island, the stimulation (played from the same loudspeaker) switched to the “target” frequency (Figure 1B). Two gerbils and two mouse lemurs (see below) were trained to perform this task in this configuration.

For gerbils, background and target frequencies of 20,000 and 660 Hz were chosen, respectively (see Supplementary Video 1). Both gerbils reached similarly high proportions of correct trials within three training sessions (Figure 1D; see figure legend for trial numbers). The percentage of successful trial completion highly exceeded chance performance levels (i.e., random stopping in the arena for > 6 s, Figures 1C,D, *P* = 6E-17 for gerbil 1 and *P* = 2E-27 for gerbil 2, binomial test, calculated for the last session). Chance performances were calculated by the use of bootstrapping methods with surrogate target locations and the actual animal locomotion trajectories (see Supplementary Figure S1A and section “Materials and Methods”). Thus, the animals stopped and remained significantly longer in the portion of the arena that triggered the appearance of the target frequency compared to any other location. This behavior was independent of the relative location of the target island position, within the arena as the animals explored the arena uniformly (i.e., no center avoidance was observed, Supplementary Figures S1B,C). Indeed, performance levels of both gerbils was significantly above chance level already for the second half of trials in the very first session of exposure to the task, and further increased with more training (Figure 1D, significance is denoted by the lower bound of the confidence interval not extending to chance level).

In both animals, more than half of the correct trials had durations of less than 30 s (half of the maximally allowed duration, inset in Figure 1D), suggesting that the chosen maximum trial length was adequate for the animals to complete the task. As rodents may exhibit history-biased behavior in operant conditioning paradigms (Busse et al., 2011), it raises the question if the gerbils might preferentially re-visit (or alternatively avoid) the locations in the arena which triggered the target stimulus in the previous correct trial. To test if they employed specific spatial bias in their search strategy based on the successful detection of the target island location in the prior trial, we plotted the linear distance between the target islands in two consecutive successful trials as a function of the time to completion in the latter of the two trials (Figure 1E). Across the two animals, no significant correlation was observed (Figure 1E, Pearson correlation, details in figure legend), demonstrating that the animals’ exploration behavior was not influenced by the short-term history of task success.

The results so far demonstrated the suitability of aSIT_freq_ for assessing frequency-change detection (discrimination) in gerbils. Next we asked to what extent these results are qualitatively specific to the innate locomotion behavior and learning capabilities of the species/clade we used (gerbils/rodentia) or generalizable across clades. To this end, we also trained two gray mouse lemurs on aSIT_freq_. Gray mouse lemurs are primates, yet comparable in size to gerbils. Notably, they exhibit a quite distinct innate exploration behavior compared to gerbils, as they usually show low levels of spontaneous exploration in an open field setting (Picq, 2016). Only once they learnt that active exploration of the setup was occasionally rewarded, exploration rate increased. Therefore, we adapted some SIT parameters accordingly and started the training with a large target island size (diameter = 32 cm), a short sit-time (1 s) and a long maximum trial duration (120 s) to increase the initial likelihood of rewarded trials. Once exploration activity of a given individual had increased, parameters were successively changed toward the target values (target diameter = 24 cm, SIT-time = 5 s, maximum trial duration = 60 s). We further introduced an initiation platform for the mouse lemurs, which allowed the animals to decide when to start a trial by visiting the platform (Figure 1F, see Supplementary Video 2, section “Materials and Methods” and section on sound localization below). Mouse lemur 1 reached the final target parameters in session 14 (after 438 trials), mouse lemur 2 in session 19 (after 567 trials). Under these conditions, both mouse lemurs achieved highly significant performance levels in aSIT_freq_ (Figure 1G, *P* = 2E-3 for mouse lemur 1 and *P* = 1E-12 for mouse lemur 2, binomial test, calculated for the last training session). Note that our surrogate island bootstrapping method to obtain chance levels and to determine significant performances (see section “Materials and Methods”) is sensitive to a subject’s moving velocity as well as the specific parameter settings of each trial and, thus, provides an objective evaluation. Hence, SIT can readily be adapted to different species.

### Multiple Island aSIT_freq_

The results so far imply that the animals’ behavior in SIT serves to seek out the target sound. However, it is unclear whether this behavior is based simply on change-detection (i.e., simply stopping whenever the stimulation changed) or if SIT can also be utilized to test the animals’ sensitivity for identification of the target stimulus. To test this hypothesis in more detail, we extended the paradigm design of SIT.

We implemented a version of aSIT_freq_ with several islands simultaneously offered in the arena (Figure 2A, see also Supplementary Video 3). The same two gerbils that were tested in the single-island task were used in this task. Four islands were simultaneously and pseudo-randomly positioned in each trial corresponding to different stimulus frequencies, including the original target frequency (660 Hz). The frequencies of the non-target islands were 460, 860, 1060, and 1320 Hz. The background frequency “outside” of islands remained as before (20,000 Hz). Importantly, in this SIT version, animals again only received a reward for sit-time stays in the actual target island (no reward was provided for sit-time stays in the non-target islands, and trials were allowed to continue). Overall, the animals showed high success rates (Figure 2B, comparable to those in aSIT_loc_, Figure 4B) already from session 1, yet because non-target island sit-time stays did not trigger trial termination, the animals could have stopped in any of the non-target islands for 6 s before entering the target island and finishing the trial. Such behavior would still correspond to a non-selective searching behavior based on detection of a change from the background frequency. Note that in this multiple island configuration of SIT, it is not possible to compute the chance level as the surrogate islands would overlap with the non-target ones which correspond to a change in frequency. To address the specificity of island preferences (and therefore the possibility of oddball strategies) directly, we calculated “sit-time incidences” *a posteriori*, that is, we determined the first island in which the animal remained for longer than the sit-time for each trial. Each recorded trial was assigned to only one island (if any at all), namely the one where the animal first stayed for longer than the sit-time. Afterward, we computed the proportion of trials that corresponded to each island frequency relative to the animal’s recurrent random sitting behavior calculated as the chance level in the last single island session (i.e., a proxy for the sit-time incidences outside of islands, see section “Materials and Methods”). Notably, significantly high sit-time incidence percentages for the target island were observed already after the first session of exposure to the multi-island aSIT_freq_ (Figure 2C, significance is given by the fact that chance level lies outside the 95% confidence interval for the target). Likewise, sit-time incidences for non-target islands dropped in prevalence after the first training session and reached baseline level for most non-target frequencies besides 860 Hz (see below). These results strongly indicate that the animals learn to specifically associate the target island frequency with the reward. It is further evidence that the animals were actively searching for the location of the target island (i.e., the arena location that induces the appearance of the target stimulus) and not simply awaiting a change in stimulation that is independent of their own spatial behavior. This assessment is further corroborated by the finding that gerbils adapted their arena occupancy during exploration according to target island location biases (see section on sound localization and Supplementary Figure S5).

Interestingly, the proportion of sit-time incidences in non-target islands was not uniform. We observed that sit-time incidences for the 860 Hz island were significantly increased relative to baseline for either animal for some of the training sessions (for gerbil 1, the lower bound of the confidence interval remained above chance level on all sessions, while for gerbil 2 it only did so on the second session). Gerbils are generally capable of discriminating even smaller frequency differences than used here (0.4 octaves) when presented in succession (Klinge and Klump, 2009). However, Chen et al. (2019) have recently shown that when confronted with a memory-based frequency discrimination task, mice generalize auditory stimuli. Therefore, one plausible explanation to the increased sit-time incidences for 860 Hz is that the gerbils generalized the new presented stimulus initially after introduction of the non-target islands.

The data, thus, suggest that multi-island SIT might represent an adequate behavioral readout of perceptual thresholds. This premise is further supported by the observation that the sit-time incidence percentage for the 860 Hz island of gerbil 2 decreased to baseline at later training sessions, which is indicative of increased frequency identification ability with experience (Figure 2C, lower panel), which could be explained by an extinction of the prior generalization (Chen et al., 2019). The reason why generalization (and extinction) is seen at 860 Hz, but not 460 Hz might be related to asymmetrical filter broadening and/or the closer logarithmic spacing (Schnupp et al., 2011).

To directly describe performance levels and their change across training sessions, we next calculated the “conditional sit-time incidences” for each of the tested island frequencies (expressed in octave distance to the target frequency - Figure 2D). For this analysis, we only considered trials where the animal encountered at least 1 s of sound exposure in the respective island, to ensure that the animal had the opportunity to evaluate the nature of the frequency change (see section “Materials and Methods”). The results of this analysis revealed two findings: first, a clear dependence of the conditional sit-time incidences on the octave-distance to the target frequency is apparent; second, the peak performance values increased, while conditional sit-time incidences of non-target frequencies decreased over the training sessions. These results indicate that learning occurred, which resulted in better identification of the different frequencies. Hence, multi-island SIT in combination with sit-time incidence analyses allows constructing psychometric functions to determine perceptual learning progress.

So far, we established that SIT allows the investigation of auditory frequency discrimination and identification in rodents and in primates. Next, we tested the suitability of SIT to study another sense, namely vision.

### Visual Grating Orientation Discrimination (vSIT_ori_)

Here, SIT was incorporated into an existing free-navigation visual stimulation setup (from Del Grosso et al., 2017, 2019) and two gerbils were trained to report when the orientation of the grating projected on the walls of the arena changed from vertical to horizontal (Figure 3B and Supplementary Video 4). Both gerbils achieved a performance above chance level (Figure 3C, *P* = 2E-28 for gerbil 3 and *P* = 1E-4 for gerbil 4, binomial test, calculated for the last training session) at the end of the training (gerbil 3 was trained in a total of 24 sessions – 672 trials – and gerbil 4 in 15 sessions – 384 trials).

Gerbil 3 was additionally tested for stimulus feature specificity by introducing a non-target island. The non-target island corresponded to a 45° orientation of the grating (Figure 3B and Supplementary Video 5). As in previous versions of SIT, this island was not rewarded if the gerbil spent longer than the sit-time inside and the trial continued. To analyze the specificity of the gerbil’s behavior, we again calculated the sit-time incidence percentage and assigned each trial to the island in which the animal stayed first for the duration of the sit-time. Already in the first session in which the non-target island was introduced, the animal exhibited high selectivity for the target stimulus and stayed for the sit-time almost exclusively in the target island (Figure 3D). The sit-time incidence percentage for the non-target island is not different from chance, which supports the hypothesis that the gerbil learned that a specific grating orientation is associated with reward and not any change in orientation. Thus, SIT is readily adaptable to other sensory modalities, suggesting that it is suitable for multi- or cross-modal investigations.

Next, we examined how SIT can be utilized to study another fundamental auditory computation – sound localization – and to what extent employing SIT (hence introducing its inherent ecological relevance by allowing free exploration) in chronically implanted animals may facilitate the identification of new neural processing signatures.

### Sound Localization (aSIT_loc_)

We applied SIT to study sound localization in freely behaving and engaged animals. Traditionally used paradigms to study spatial sensitivity require a constant head position during sound presentation (Wood et al., 2019), often in naïve or anesthetized animals (Middlebrooks and Knudsen, 1984). In contrast, aSIT_loc_ allows investigations in the locomoting animal during active localization, providing more naturalistic conditions and, thus, higher ecological relevance. We used the single-island configuration, yet here the target island cue was a change in the sound source location (i.e., the active loudspeaker). The arena was equipped with two diametrically opposed loudspeakers (180° angle separation from the center of the arena), from which a short (57 ms) harmonic complex sound (see section “Materials and Methods”) was presented at 4 Hz repetition rate. Upon trial initiation (see below), the sound was played by one of the two loudspeakers (the background) until the animal entered the target island, at which moment the stimulation switched to the second loudspeaker (target) (Figure 4A and Supplementary Video 6). The identity of the target and background loudspeaker was maintained throughout training and testing yet catch-trials with swapped identities were introduced in a subset of the animals (see below). Since we combined this paradigm with neural recordings in the auditory cortex (AC), we added an initiation platform (∼1 cm in height) for the animals during training and testing on aSIT_loc_ (similar to the mouse lemur paradigm in aSIT_freq_). Voluntary trial initiation has been shown to reduce spontaneous discharge and improve the detection of thresholds (Buran et al., 2014) and task engagement sharpens spatial tuning of neurons in AC in cats (Lee and Middlebrooks, 2011). The platform was positioned near the wall of the arena and animals were required to stay on it for one second to start a trial.

### Locomotion and Sitting Behavior Are Specific to Target Loudspeaker and to Target Island Distribution Likelihood

We tested 11 gerbils in aSIT_loc_, all of which reached highly significant success rates (Figure 4B, *P* = 0.0033, *N* = 11 gerbils, Wilcoxon signed-rank test). Swapping the identity of the target and background loudspeakers in 1/8 of trials during the testing phase (the identities of target and background loudspeakers remained fixed during training) resulted in performance levels that were significantly lower than chance level (Figure 4B, *P* = 0.018, *N* = 7 gerbils, Wilcoxon signed-rank test). Given that these catch-trials started with the presentation of the usual target stimulus, the animals could potentially have just stopped moving immediately after initiating a trial in anticipation of the reward, which could explain the extremely low success rate. However, further analysis revealed that the animals indeed encountered the target-islands with similar prevalence in catch-trials as in normal trials, but rarely remained in the island for the required sit-time in catch-trials (Supplementary Figure S4). Thus, the animals actively avoided staying in the target island in these catch-trials, revealing that they indeed associated the identity of the active loudspeaker (target or background) with reward predictability. Since the spatial location of the active loudspeaker was the only parameter that allowed determination of loudspeaker identity, these data validate that the animals were actively localizing the sound source to achieve task performance. Hence, similar as for frequency discrimination, the gerbils did not follow an oddball strategy but specifically searched for the target stimulus.

We also tested to which extent the animals associate their locomotive searching behavior with target detection success. To this end, we employed a biased distribution likelihood of target island locations in the arena. We found that after the animals were trained on one specific distribution likelihood, their arena occupancy was specific to this distribution (Supplementary Figure S5). That is, the animals predominantly visited locations in the arena that were most likely to contain the target island. Thus, a clear association existed between the animals’ locomotive behavior and their reward expectancy, i.e., they actively searched for the target island position. Together, these data validate that SIT allows the interrogation of different cues based on the concept of a locomotive search for a target stimulus (i.e., island).

### Electrophysiological Recording of Neural Activity During SIT Performance

We were interested in combing SIT with chronic electrophysiological recording techniques. Specifically, we asked to what extent the unrestricted self-movement and task relevance that are provided by SIT might facilitate exploring neural signatures of spatial processing in AC. Therefore, we implanted a tetrode bundle in AC of a previously trained gerbil (see section “Materials and Methods”), and recorded brain activity during task performance in aSIT_loc_. We collected local field potential (LFP), from which we calculated Auditory Evoked Potentials (AEPs). Remarkably, although the acoustic stimulation was identical from both loudspeakers (sound intensity was roved throughout trials), AEPs were different between the two sound sources (Figures 4C,D). Specifically, AEP amplitudes were significantly larger during stimulation by the target loudspeaker (*P* = 0.000049, Mann-Whitney *U*-test). A plausible reason for this difference in AEP amplitude could be differences in the intensity of the sounds presented from each loudspeaker, due to the animal being closer to the target loudspeaker than to the non-target, at the moment of respective sound presentation. This does not seem to be the case, as the histograms of animal position for target and non-target loudspeaker sound presentations do not show such a bias (Supplementary Figure S6). More likely, these data suggest that the learned relevance of each specific sound source modulates neural response amplitude. Such differences in sound-source-specific responses have – to our knowledge – not previously been reported in studies on spatial processing and thus demonstrate that the use of SIT may be beneficial to reveal neuronal signatures of sensory processing under ecologically relevant conditions.

## Discussion

SIT is a novel experimental paradigm for freely moving animals that are actively engaged in a sensory processing task and can be combined with simultaneous neural recordings. It exploits voluntary exploratory self-motion – and its cessation upon detection of a change in the sensory stimulation – for testing psychophysical sensitivity in a variety of cues and sensory modalities. Self-motion occurs constantly under natural conditions and, throughout evolution, neural processing has adapted to the resulting continuous modulation of the sensory input (Niell and Stryker, 2010; Zhou et al., 2014; McGinley et al., 2015; Williamson et al., 2015; Willett et al., 2019). SIT consequently captures ethologically relevant behavior that is crucial for sensory processing and decision making. SIT was inspired by existing closed-loop free navigation assays (Polley et al., 2004; Whitton et al., 2014), but differs significantly in a number of aspects. Most importantly, the introduction of discrete sensory islands instead of a gradient fundamentally changes the locomotion behavior toward free exploration of the entire arena. Moreover, the introduction of multiple islands allows the interrogation of animals about perception thresholds and the construction of psychometric functions.

The last decade has seen a rise in the study of perceptual decision making, particularly in rodents. Data from established and commonly used paradigms, such as go/no-go tasks (G/NG) and two alternative forced choice tasks (2AFC), can be difficult to interpret. For example, in 2AFC designs, the animals are forced to give an answer on every trial, which renders the disentanglement between real decisions and guesses difficult (Carandini and Churchland, 2013). The sensory environment in which rodents are immersed while performing these tasks has been increasing in complexity in recent years, from lever operation, to full 360° virtual reality with online locomotive update. However, animals require substantial training to learn how to use and navigate these setups. Moreover, a major drawback of many virtual reality setups is a lack of vestibular feedback (due to head fixation) that is naturally present during self-movement.

In contrast, SIT is characterized by shorter training periods than many traditional behavioral paradigms or techniques involving virtual reality (e.g., as little as one training session for gerbils in aSIT_freq_), high flexibility to readily adapt parameters to both the constraints of the scientific question at hand and to the behavioral characteristics of the animal clade used. If required (e.g., depending on complexity and species), the motivational state of the animals can be controlled by addition of an initiation platform, which assures the willingness of the individual to perform a trial. In essence, SIT represents a refined version of a G/NG task. Nonetheless, the possibility to add multiple non-target islands allows testing of cue identification and determining psychometric functions. In its currently presented form with pseudo-randomized island locations, SIT does not represent a spatial association nor a long-term memory task. Nonetheless, SIT can be easily transformed into such a task by maintaining the target island location constant across trials or switching between a limited number of target locations; e.g., a recent study by Rossato et al. (2018) which used electromagnets to switch between available islands in the Morris water maze could be performed in SIT, with greater flexibility due to the amount and position of the islands depending on software rather than hardware. In addition, the lack of water in SIT facilitates maintenance of the setup and coupling of experiments with interventions such as electrophysiology. Although dry versions of the water maze already exist, such as in Bast et al. (2005), where animals forage for food in hidden compartments, SIT provides an easier, more versatile alternative in which the search for food can be replaced by the search for target island (to receive food reward). Thus, spatial learning and memory studies in relation to sensory cuing could be performed, a task of high ecological relevance in many species (Sherry, 1985; Collett et al., 1986).

In any of its potential variants, combining SIT with specific time points of electrode implantation (e.g., before/during training), opens exciting possibilities to study aspects of learning and plasticity of sensory processing during voluntary self-motion and active sensing. We have exemplified some of this potential here, as our AC recording during aSIT_loc_ revealed previously unreported response modulation of spatial sensitivity based on sound source identity. Previous reports had established that neuronal responses in auditory cortex can be modulated by “attention” (Hubel et al., 1959; Evans and Whitfield, 1964). Our findings are related, but potentially more profound, as the difference in responses to both loudspeakers is unlikely to be due to the attentive state of the animal, but rather the relative relevance of the two sound sources regarding reward expectancy and experimental design. Multiple studies in AC have found relevance-specific response modulation in animals if engaged in the experimental task (Miller et al., 1972; Fritz et al., 2003, 2007; Atiani et al., 2009; Otazu et al., 2009; Lee and Middlebrooks, 2011; Guo et al., 2019). Moreover, a recent study with macaque monkeys that were trained to respond differentially to the same auditory stimulation depending on the context reported larger auditory cortex responses to the same stimulus when it required a no-go response (Huang et al., 2019). Likewise, greater neural responses during aSIT_loc_ were observed for target sounds that required the animal to remain sitting.

In summary, SIT is a flexible and easily implementable behavioral paradigm that uniquely incorporates self-motion and natural exploratory behavior, which are essential for ecological sensory processing. SIT is readily applicable across species and sensory modalities and extendable to use for neurophysiological investigations. Beyond the options we have exemplified here, SIT is widely adaptable to a large variety of neuroscientific and ecological fields. For example, besides the auditory and visual cues probed here, we suggest that somatosensory cues can be studied by dynamically changing the floor texture, or olfactory sensitivity could be tested collocating the target island and odor release valves beneath the arena. Similarly, decision-making based on congruent or ambiguous combinations of different sensory modalities is ecologically important and could readily be applied in SIT. In the future, it would be particularly interesting to use high yield recording devices, such as neuropixel electrodes (Juavinett et al., 2019), to sample a wide range of brain areas. Moreover, the ongoing miniaturization of technology will allow precise stimulus control in various sensory modalities and combinations (e.g., through wireless miniature cameras or microphones). These new technologies coupled with SIT should garner unprecedented insights to unravel ecologically relevant sensory neural processes.

## Data Availability Statement

The raw data supporting the conclusions of this article will be made available by the authors, without undue reservation, to any qualified researcher.

## Ethics Statement

All procedures involving gerbils were reviewed and approved by the Regierung von Oberbayern AZ 55.2-1-54-2532-74-2016 and AZ 55.2-1-54-2532-70-2016. All procedures involving mouse lemurs were reviewed and approved by the Animal Welfare Committee of the LAVES (AZ 33.19-42502-04-18/3050).

## Author Contributions

MP, DNF, DA, and LB conceived SIT. MP, DNF, and DA designed the experiments. BG, DS, and ASi contributed to paradigm refinement. DA, DNF, DS, ASo, and PG performed the experiments. LB, DA, DS, and ASo contributed to programming SIT code. DA, DNF, DS, and ASo analyzed the results. DNF, MP, and DA designed and generated the figures and wrote the manuscript. All authors provided comments and approved the manuscript.

## Conflict of Interest

The authors declare that the research was conducted in the absence of any commercial or financial relationships that could be construed as a potential conflict of interest.

## Dedication

This work is dedicated to Lutz Wiegrebe, whose expertise and help was invaluable for the development of SIT.

## Supplementary Material

FIGURE S1 **(A)** Schematic representation of the surrogate island random permutation. Colored line depicts a real trajectory of an animal in a trial color coded with the time at which the animal was at each position, starting from the initiation platform (filled gray circle). The real target island is where the animal ends (open orange circle). The dots correspond to the position of the animal with 1s interval between them. The chance level of task completion was calculated using *a posteriori* surrogate island locations (open black circles, only a few shown here from the 1000 actually used for each trial). **(B)** Trajectories of gerbil 1 and 2 during the 2nd training session separated by correct and incorrect trials: no apparent change in pattern of locomotion is seen when the animal did not succeed in the task. **(C)** Comparison of the distance of the target island’s center to the center of the arena between correct and incorrect trials for gerbil 1 and 2 in the same session as in B. Gerbil 1: N_correct_ = 44, N_incorrect_ = 28, P = 0.12; Gerbil 2: N_correct_ = 44, N_incorrect_ = 21, P = 0.63 (Mann-Whitney U test). Boxplots depict the median (black line), 1st and 3rd quartile (filled boxes), ± 2.7 σ (whiskers) and outliers (cross).

FIGURE S2 Duration of correct trials in aSIT_freq_ for gerbils (left panel) and for mouse lemur (Right panel).

FIGURE S3 Mouse lemur performance at intermediate training sessions, relative to chance level. Target island diameter = 26.7 cm. For mouse lemur 1, x = 6 and sit-time = 4 s. For mouse lemur 2, x = 5 and sit-time = 2 s.

FIGURE S4 Comparison in the aSIT_loc_ version between the trials in which the target loudspeaker was the one from the training, with catch-trials (1/8 of total trials) in which the opposite loudspeaker was the target one. **(A)** The gerbils found the target island as often in catch-trials as in normal target trials. **(B)** The gerbils left the target island much more often (∼85% trials) in catch-trials than in normal target trials (∼35% trials). Only situations where the gerbils stayed in the target island for at least 1 s were used to assure the gerbil listened to the sound and did not just run through the island. Number of sessions: 39; Number of normal target trials: 1784; Number of catch trials: 285. Uncertainty was determined using a bootstrapping method.

FIGURE S5 Association between spatial position and stimulus change in the aSIT_loc_. **(A)** Distribution of the target islands for all the trials in a session where there was not a target location bias (left) and in a session where there was a target location bias (right). The filled gray circle corresponds to the initiation platform. The dashed magenta circle radius is twice as large as that of a target island and divides the target islands which were considered to be in the center (light gray circles) from the target islands considered not to be in the center (dark blue circles). In sessions without target location bias ∼59% of the islands occurred in the center whereas, in sessions with target location bias, ∼78% occurred in the center. **(B)** Difference in percentage of successful trials between trials in which the target was in the center and trials in which the target was not in the center (error bars correspond to the 95% confidence interval, calculated using a bootstrapping method). Gerbil 1 and 2 (these are not the same gerbils that were trained in aSIT_freq_) were first trained in an unbiased condition and the bias condition was later introduced. Gerbil 3 was first trained in a biased condition, and the bias was later removed. When the target location was biased to the center, the animals spent more time in that region and their performance increased in relation to when the target was outside the center.

FIGURE S6 Histograms of gerbil position at sound presentation times for the session during which LFP was recorded, reported on main (Figure 4). Left panel shows the histogram for target stimulus presentations (orange loudspeaker). Right panel shows the histogram for background stimulus presentations (blue loudspeaker).
