# Supplementary figures and images for: Sensory Island Task (SIT): A New Behavioral Paradigm to Study Sensory Perception and Neural Processing in Freely Moving Animals
Source: Front Behav Neurosci. 2020 Sep 25;14:576154. doi: 10.3389/fnbeh.2020.576154 (PMC7546252; doi:10.3389/fnbeh.2020.576154)

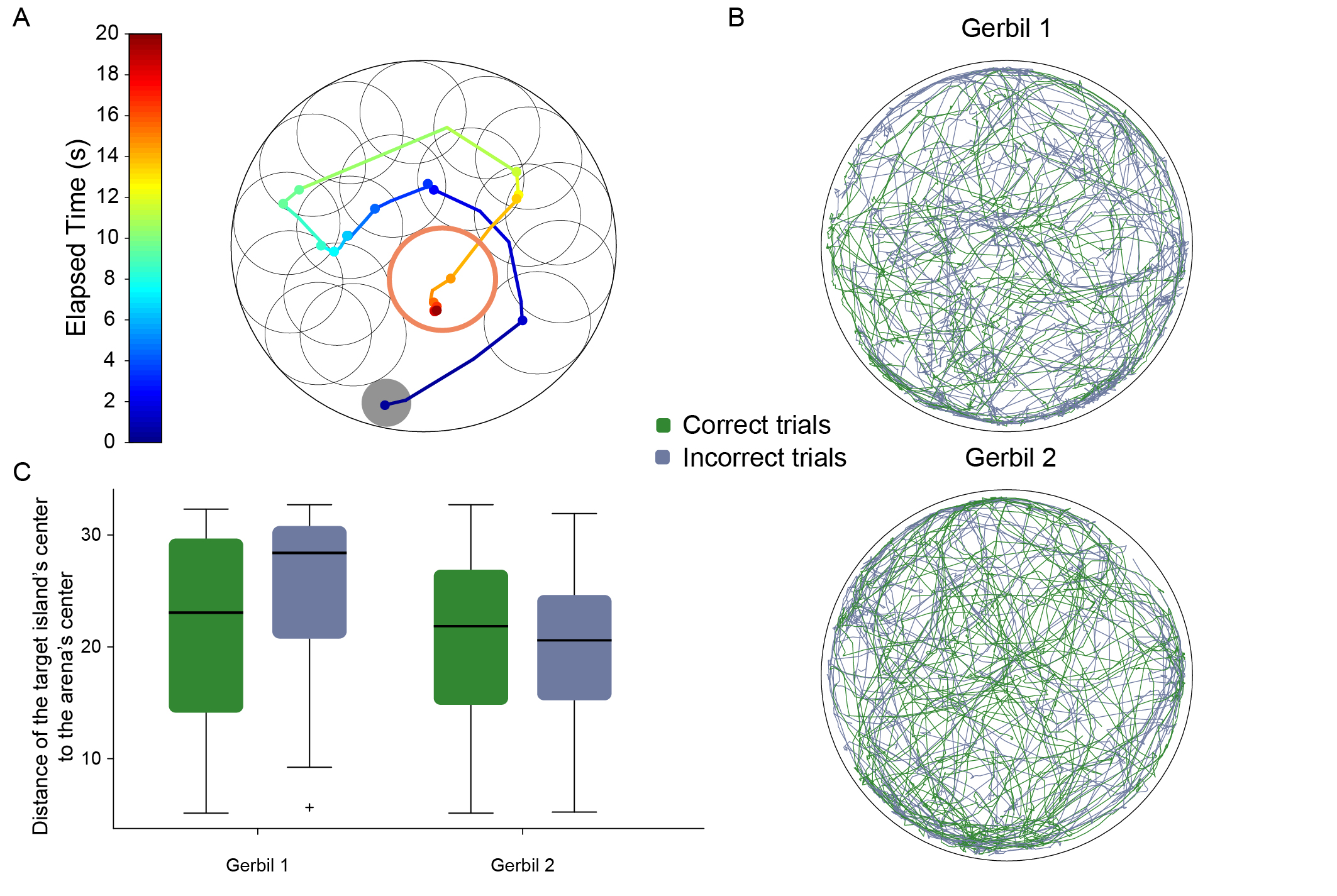

Supplement: FIGURE S1 — (A) Schematic representation of the surrogate island random permutation. Colored line depicts a real trajectory of an animal in a trial color coded with the time at which the animal was at each position, starting from the initiation platform (filled gray circle). The real target island is where the animal ends (open orange circle). The dots correspond to the position of the animal with 1s interval between them. The chance level of task completion was calculated using a posteriori surrogate island locations (open black circles, only a few shown here from the 1000 actually used for each trial). (B) Trajectories of gerbil 1 and 2 during the 2nd training session separated by correct and incorrect trials: no apparent change in pattern of locomotion is seen when the animal did not succeed in the task. (C) Comparison of the distance of the target island’s center to the center of the arena between correct and incorrect trials for gerbil 1 and 2 in the same session as in B. Gerbil 1: Ncorrect = 44, Nincorrect = 28, P = 0.12; Gerbil 2: Ncorrect = 44, Nincorrect = 21, P = 0.63 (Mann-Whitney U test). Boxplots depict the median (black line), 1st and 3rd quartile (filled boxes), ± 2.7 σ (whiskers) and outliers (cross). [file Image_1.jpg]

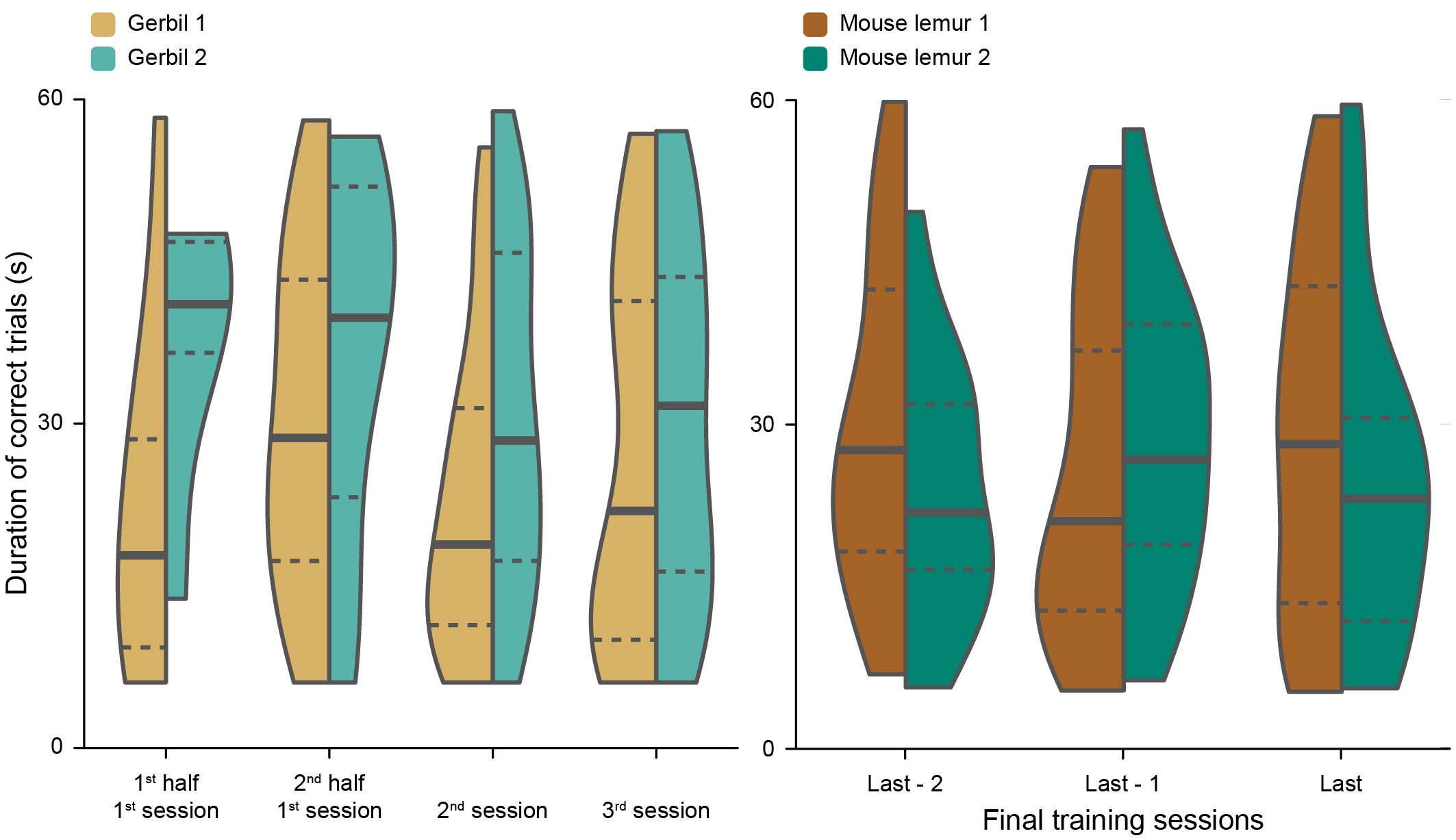

Supplement: FIGURE S2 — Duration of correct trials in aSITfreq for gerbils (left panel) and for mouse lemur (Right panel). [file Image_2.jpg]

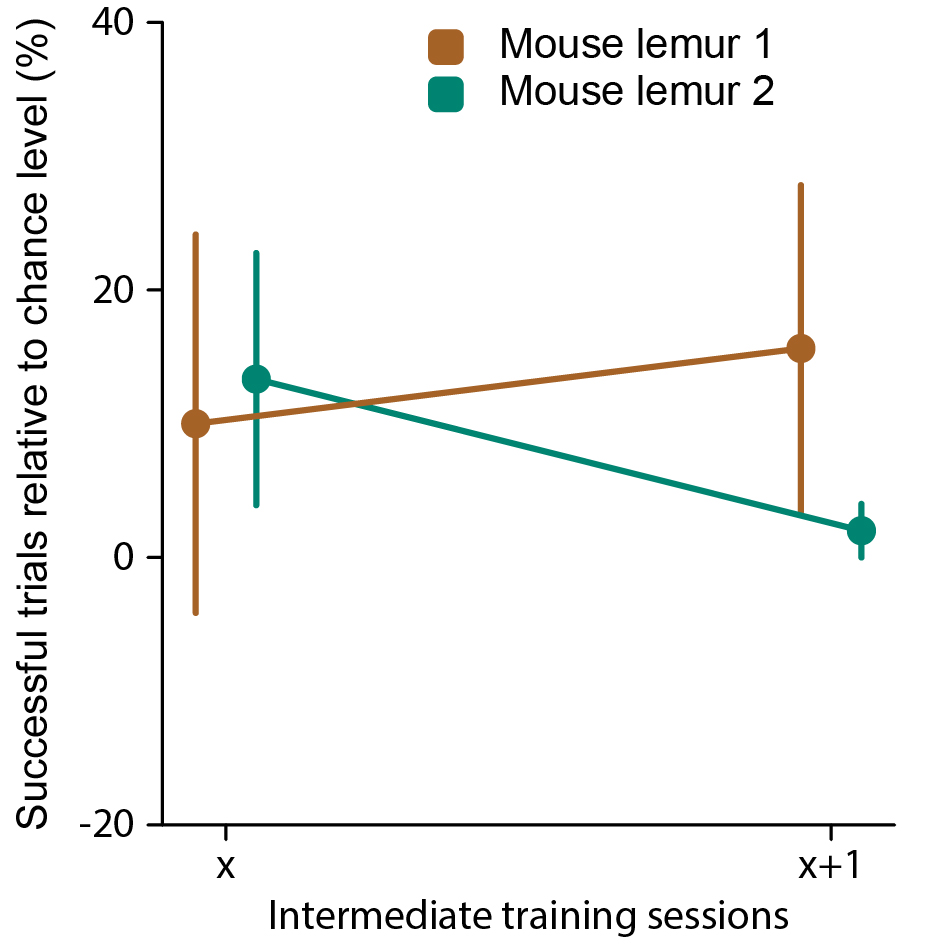

Supplement: FIGURE S3 — Mouse lemur performance at intermediate training sessions, relative to chance level. Target island diameter = 26.7 cm. For mouse lemur 1, x = 6 and sit-time = 4 s. For mouse lemur 2, x = 5 and sit-time = 2 s. [file Image_3.jpg]

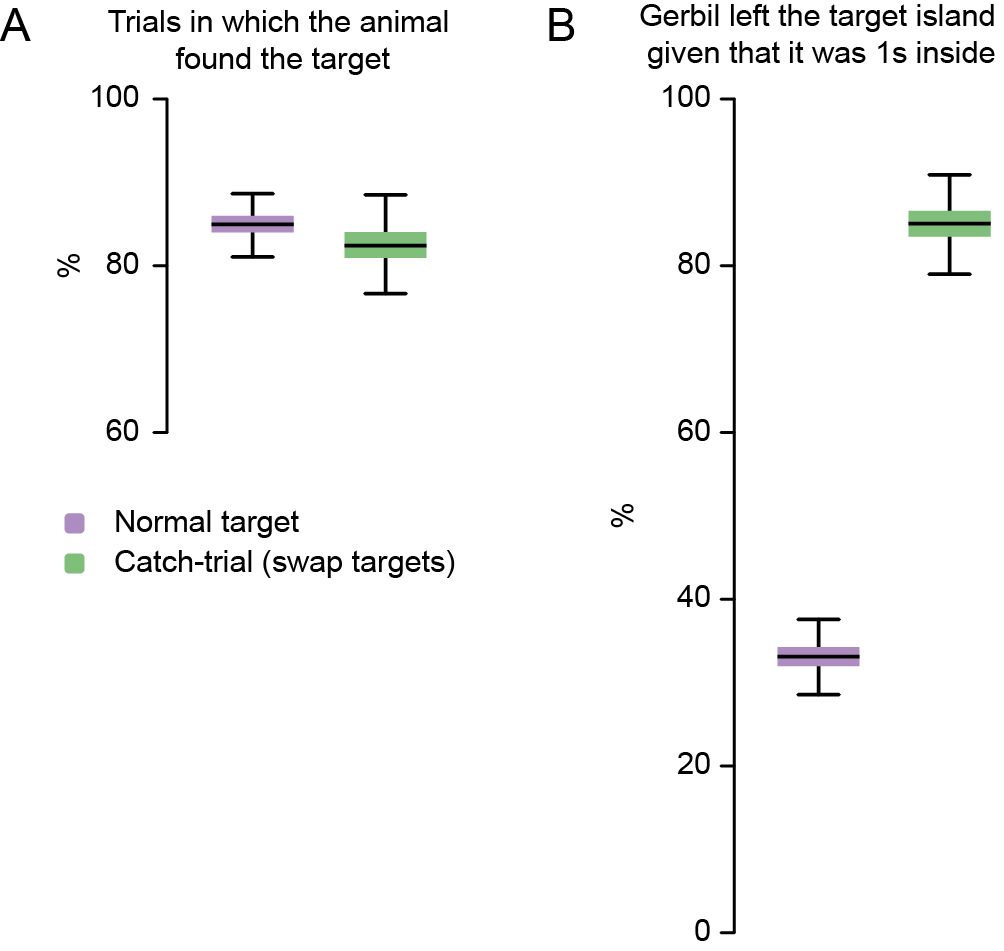

Supplement: FIGURE S4 — Comparison in the aSITloc version between the trials in which the target loudspeaker was the one from the training, with catch-trials (1/8 of total trials) in which the opposite loudspeaker was the target one. (A) The gerbils found the target island as often in catch-trials as in normal target trials. (B) The gerbils left the target island much more often (∼85% trials) in catch-trials than in normal target trials (∼35% trials). Only situations where the gerbils stayed in the target island for at least 1 s were used to assure the gerbil listened to the sound and did not just run through the island. Number of sessions: 39; Number of normal target trials: 1784; Number of catch trials: 285. Uncertainty was determined using a bootstrapping method. [file Image_4.jpg]

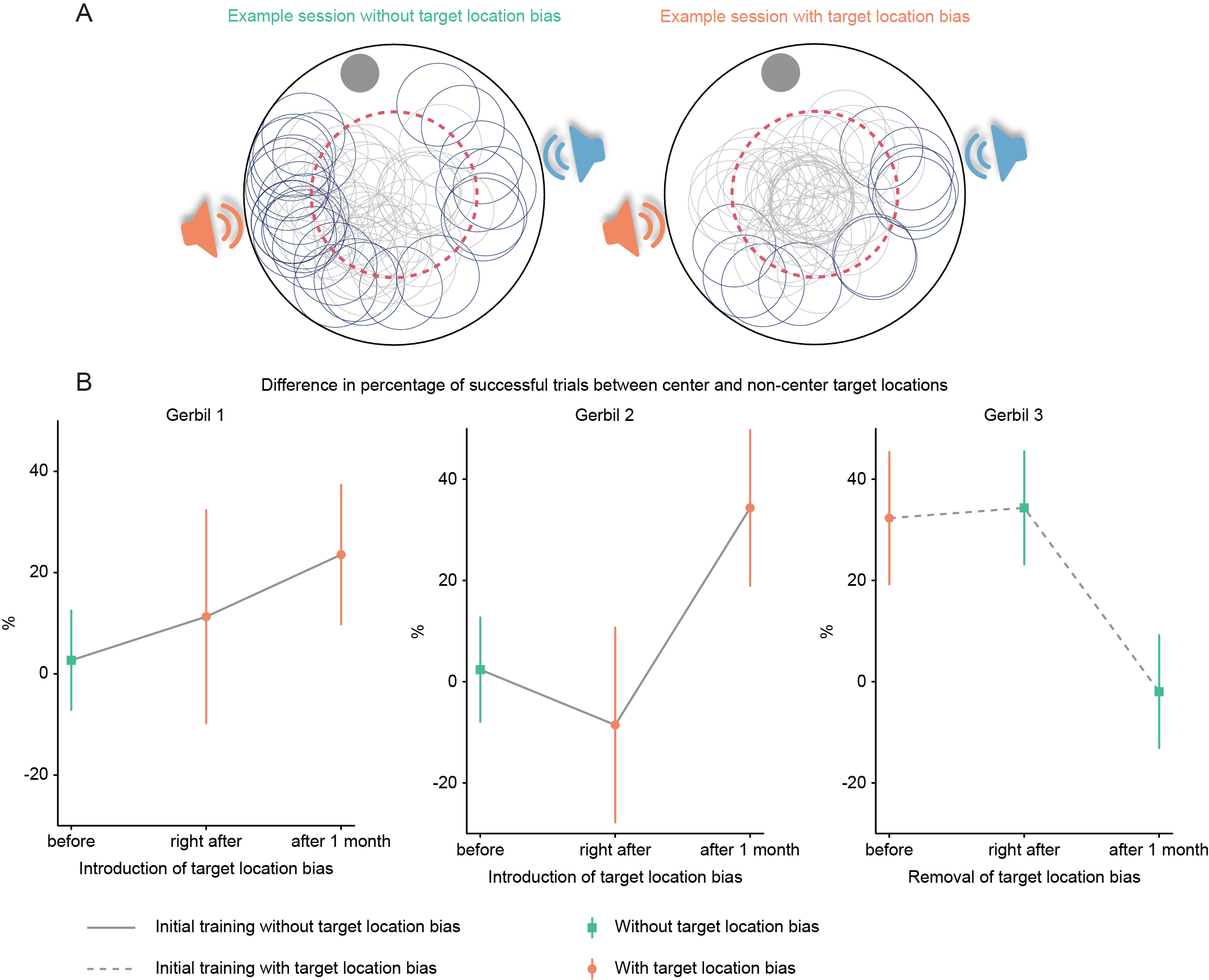

Supplement: FIGURE S5 — Association between spatial position and stimulus change in the aSITloc. (A) Distribution of the target islands for all the trials in a session where there was not a target location bias (left) and in a session where there was a target location bias (right). The filled gray circle corresponds to the initiation platform. The dashed magenta circle radius is twice as large as that of a target island and divides the target islands which were considered to be in the center (light gray circles) from the target islands considered not to be in the center (dark blue circles). In sessions without target location bias ∼59% of the islands occurred in the center whereas, in sessions with target location bias, ∼78% occurred in the center. (B) Difference in percentage of successful trials between trials in which the target was in the center and trials in which the target was not in the center (error bars correspond to the 95% confidence interval, calculated using a bootstrapping method). Gerbil 1 and 2 (these are not the same gerbils that were trained in aSITfreq) were first trained in an unbiased condition and the bias condition was later introduced. Gerbil 3 was first trained in a biased condition, and the bias was later removed. When the target location was biased to the center, the animals spent more time in that region and their performance increased in relation to when the target was outside the center. [file Image_5.jpg]

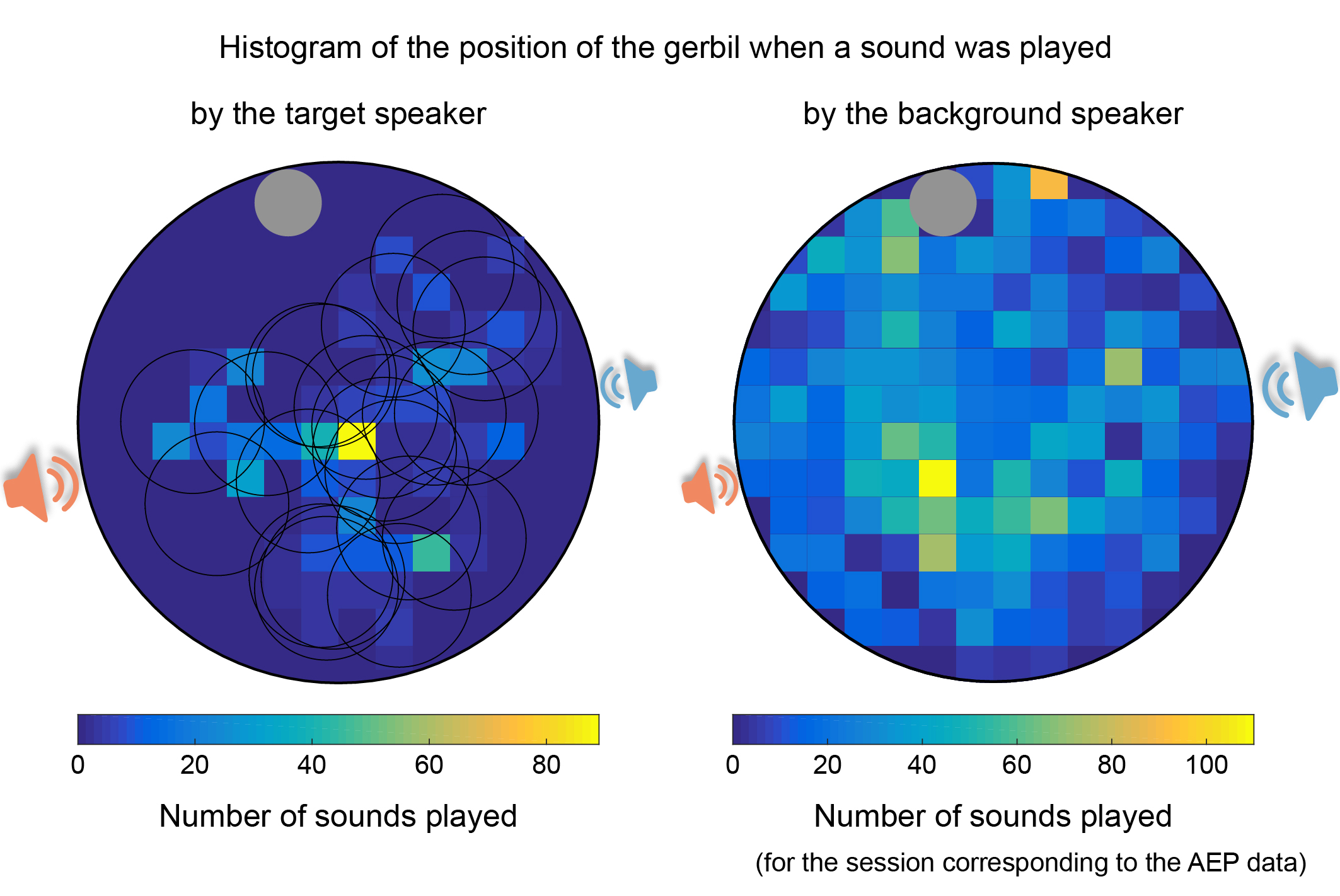

Supplement: FIGURE S6 — Histograms of gerbil position at sound presentation times for the session during which LFP was recorded, reported on main (Figure 4). Left panel shows the histogram for target stimulus presentations (orange loudspeaker). Right panel shows the histogram for background stimulus presentations (blue loudspeaker). [file Image_6.jpg]
